# Assessment of Digital Capabilities by 9 Countries in the Alliance for Healthy Cities Using AI: Cross-Sectional Analysis

**DOI:** 10.2196/62935

**Published:** 2025-02-07

**Authors:** Hocheol Lee

**Affiliations:** 1 Department of Health Administration, College of Software and Digital Healthcare Convergence, Yonsei University, PO Box 26493Wonju, Repubic of Korea

**Keywords:** digital capabilities, digital health cities, digital transformation, Asian Forum of Healthy Cities, assessment, digital health, artificial intelligence, AI, World Health Organization, WHO, healthy city, data, health management, digital era, qualitative analysis, cross-sectional survey, database, digital health database, effectiveness, digital literacy

## Abstract

**Background:**

The Alma-Ata Declaration of 1978 initiated a global focus on universal health, supported by the World Health Organization (WHO) through healthy cities policies. The concept emerged at the 1984 Toronto “Beyond Health Care” conference, leading to WHO’s first pilot project in Lisbon in 1986. The WHO continues to support regional healthy city networks, emphasizing digital transformation and data-driven health management in the digital era.

**Objective:**

This study explored the capabilities of digital healthy cities within the framework of digital transformation, focusing on member countries of the Asian Forum of Healthy Cities. It examined the cities’ preparedness and policy needs for transitioning to digital health.

**Methods:**

A cross-sectional survey was conducted of 9 countries—Australia, Cambodia, China, Japan, South Korea, Malaysia, Mongolia, the Philippines, and Vietnam—from August 1 to September 21, 2023. The 6-section SPIRIT (setting approach and sustainability; political commitment, policy, and community participation; information and innovation; resources and research; infrastructure and intersectoral; and training) checklist was modified to assess healthy cities’ digital capabilities. With input from 3 healthy city experts, the checklist was revised for digital capabilities, renaming “healthy city” to “digital healthy city.” The revised tool comprises 8 sections with 33 items. The survey leveraged ChatGPT (version 4.0; OpenAI, Microsoft), accessed via Python (Python Software Foundation) application programming interface. The *openai* library was installed, and an application programming interface key was entered to use ChatGPT (version 4.0). The “GPT-4 Turbo” model command was applied. A qualitative analysis of the collected data was conducted by 5 healthy city experts through group deep-discussions.

**Results:**

The results indicate that these countries should establish networks and committees for sustainable digital healthy cities. Cambodia showed the lowest access to electricity (70%) and significant digital infrastructure disparities. Efforts to sustain digital health initiatives varied, with countries such as Korea focusing on telemedicine, while China aimed to build a comprehensive digital health database, highlighting the need for tailored strategies in promoting digital healthy cities. Life expectancy was the highest in the Republic of Korea and Japan (both 84 y). Access to electricity was the lowest in Cambodia (70%) with the remaining countries having had 95% or higher access. The internet use rate was the highest in Malaysia (97.4%), followed by the Republic of Korea (97.2%), Australia (96.2%), and Japan (82.9%).

**Conclusions:**

This study highlights the importance of big data-driven policies and personal information protection systems. Collaborative efforts across sectors for effective implementation of digital healthy cities. The findings suggest that the effectiveness of digital healthy cities is diminished without adequate digital literacy among managers and users, suggesting the need for policies to improve digital literacy.

## Introduction

The Alma-Ata Declaration of 1978 marked a global shift toward universal health, a movement endorsed by the World Health Organization (WHO) through its advocacy for healthy cities as a strategic policy to enhance global health [1]. The idea of a healthy city was first conceptualized at the 1984 Toronto “Beyond Health Care” conference, and the first pilot project was spearheaded by the WHO European Office in Lisbon in April 1986 [2]. Subsequently, the International Health Cities Committee was established, and over 3000 cities now have healthy cities policies. The WHO remains a steadfast supporter of regional healthy city networks. In Europe, the WHO European Healthy Cities Network, founded in 1988, continuously promotes networking through assemblies every 5 years, involving over 1400 municipalities [3]. In North America, a network was established with Canada as the central player in 1984, which now includes over 200 North American cities. In the Western Pacific region, the Alliance for Healthy Cities (AFHC) was established in 2003. It certifies and manages healthy cities as alliance members, facilitating dynamic networking through biennial general assemblies [4]. In the Western Pacific region, 9 countries—Australia, Cambodia, China, Japan, the Republic of Korea, Malaysia, Mongolia, the Philippines, and Vietnam—are actively participating as full members of the AFHC. The AFHC is an international network focused on protecting and promoting the health of urban residents through collaborative efforts.

Healthy cities are dedicated to improving physical and social environments where all community members collaborate to enhance the health and quality of life of citizens [5]. The evaluation of healthy cities is categorized into distinct areas: personal, community, socioeconomic infrastructure, environment, and evaluation management. These domains all adopt a top-down approach, underscoring the pivotal role of policy makers in formulating and executing policies and initiatives for healthy cities [6]. Research has shown that the proactive stance of the city’s leaders has a positive impact on community residents’ health [7]. Additionally, healthy city projects must be developed considering the specific contemporary and environmental demands of the country. Worldwide, healthy cities are adopting unique characteristics, transitioning to models such as senior-friendly cities, smart healthy cities, digital healthy cities, and green healthy cities [8,9]. Following the coronavirus disease 2019 pandemic, there was an acceleration in digital transformation, a trend pivotal in the context of healthy cities. The WHO highlighted that healthy cities in the digital era should focus on data-driven monitoring and the implementation of digital platforms for managing the public’s health [10]. With the advancement of digital technology, it has become crucial for healthy cities to prioritize the development of digital competencies.

The WHO’s “Global Strategy on Digital Health 2020‐2025” emphasizes the role of healthy cities in the digital age, including citizen-led health management; policy making based on big data; and the expansion of digital platforms, including telemedicine [11]. Particularly, the strategy underscores the importance of health management through information technology devices and the significance of digital health at the national level [11]. Additionally, the AFHC stresses the need to integrate aging, smart technology, and digital solutions in healthy city strategies for the Western Pacific region. It prioritizes digital-focused strategies for advancing healthy cities [12].

The world is preparing for the digital era through evident forms of digital transformation such as digital healthy cities and smart healthy cities. Previous studies have stressed the importance of equipping nations and cities with digital competency readiness to prepare for this transformation [13]. However, according to United Nations digital indicators, there are disparities in digital capabilities among countries in the Western Pacific region and the AFHC member countries [14].

Several methods are used to measure the capabilities of healthy cities, such as the WHO European Healthy City Evaluation Model and the AFHC SPIRIT (setting approach and sustainability political commitment, policy, and community participation; information and innovation; resources and research; infrastructure and intersectoral; and training) checklist [15]. These 2 models, conceptualized and evaluated by healthy city experts, provide a structured, logical framework for objectively assessing various aspects of a city, including its health status, capabilities, lifestyle, vision, strategy, and network. Healthy cities worldwide use these models, and they have recently been used to understand the capabilities of healthy cities in the digital era.

In the context of digital transformation, this study aims to investigate the capabilities of digital healthy cities in all AFHC member countries, exploring policy implications for areas that require further development and readiness to effectively promote the transition to digital healthy cities.

## Methods

### Study Design

This cross-sectional study aims to inspect the digital healthy city capabilities among AFHC member countries. Nine countries were included: Australia, Cambodia, China, Japan, the Republic of Korea, Malaysia, Mongolia, the Philippines, and Vietnam.

### Instruments

We used a modified version of the SPIRIT checklist developed and used by AFHC to assess the capabilities of healthy cities [16,17]. The checklist consists of 6 sections: setting approach and sustainability, political commitment, policy, and community participation, information and innovation, resources and research, infrastructure and intersectoral, and training. We modified the tool with input from 3 healthy city experts to more specifically evaluate digital capabilities, particularly changing the term “healthy city” used in the original tool to “digital healthy city.” The modified tool contained 8 sections with 33 items.

### Data Collection

Data were collected using a database powered by a large-scale artificial intelligence (AI) application. ChatGPT (version 4.0), developed by OpenAI, was accessed via an application programming interface in Python and used to conduct our survey. We installed the *openai* library in Python and entered an application programming interface key issued for the use of ChatGPT (version 4.0). In the *openai* library, the “GPT-4 Turbo” model command was used; GPT-4 Turbo is a large AI language model that has acquired knowledge up to March 2023.

The query command was set up to allow the input of questions. Additionally, the “message” command was used to define the conversation’s history for generation. The system option facilitated the setup of a chatbot helper for conversation options, and messages were relayed directly to the helper via the assistant. The user option was used to save previous conversations and maintain dialogue continuity. The results were made available through the answer command.

The questions on the SPIRIT checklist were revised to reflect names of the 9 surveyed AFHC member countries. The survey was conducted from August 1 to September 21, 2023, and the collected data were stored in a report. No human participants were involved, and thus institutional review board (IRB) approval was not obtained.

### Data Analysis

Five healthy city experts performed a qualitative analysis on the collected data through a group deep discussion. The experts were recruited from October 1, 2023, to November 3, 2023. They had experience conducting activities within the AFHC International Network, held PhDs, and were affiliated with healthy city networks in their respective countries. Specifically, the group comprised 3 public health PhDs from Yonsei University’s Healthy City Research Center and two professors who are academic members of the Healthy City Society. A consultation meeting was held on November 13, 2023. Prior to the qualitative analysis meeting, written informed consent was obtained from the 5 experts; the consent covered this study’s purpose and plan and the objectives of the qualitative analysis meeting. Through this process, we obtained the experts’ advice on the appropriateness of the contents of the SPIRIT checklist.

First, the collected reports were summarized and organized based on key areas on the checklist. Second, a relational analysis was conducted of keywords from the summarized reports to visualize the results. Third, 3 digital healthy city experts conducted a final review and discussed the digital capabilities and directions for each country. The indicators for healthy cities in the 9 surveyed countries (Table 1) were analyzed using the official AFHC data, World Bank’s DataBank, and International Telecommunication Union Data Hub [14,18,19].

**Table 1.** AFHC^a^ member cities.

	AUS^b^	KHM^c^	CHN^d^	JPN^e^	KOR^f^	MYS^g^	MNG^h^	PHL^i^	VNM^j^
Number of healthy cities, n^k^	5	1	34	31	109	1	5	12	1
Population, million (year)	25.9 (2021)	16.7 (2022)	1410 (2022)	125.1 (2022)	51.6 (2022)	33.9 (2022)	3.3 (2022)	115.5 (2022)	98.1 (2022)
GDP^l^ per capita, US $ (year)	64,491 (2022)	1786 (2022)	12,720 (2022)	33,815 (2022)	32,254 (2022)	11,971 (2022)	4946 (2022)	3498 (2022)	4163 (2022)
GDP growth, annual % (year)	3.6 (2022)	1.1 (2022)	3 (2022)	−0.4 (2022)	2.6 (2022)	8.7 (2022)	4.8 (2022)	7.6 (2022)	8 (2022)
Poverty headcount ratio, % (year)	0.5 (2018)	N/A^m^	0.1 (2019)	0.7 (2013)	0.2 (2016)	0 (2018)	0.7 (2018)	3 (2018)	0.7 (2018)
Life expectancy at birth, total years (year)	83 (2021)	70 (2021)	78 (2021)	84 (2021)	84 (2021)	75 (2021)	71 (2021)	69 (2021)	71 (2021)
Access to electricity, %	100	82	100	100	100	100	100	97	100
Safely managed sanitation services, %	74	N/A	70	81	100	77	56	61	N/A
Internet use rate, %	96.2	60.2	75.6	82.9	97.2	97.4	81.6	52.7	78.6

a AFHC: Alliance for Healthy Cities.

b AUS: Australia.

c KHM: Cambodia.

d CHN: China.

e JPN: Japan.

f KOR: the Republic of Korea.

g MYS: Malaysia.

h MNG: Mongolia.

i PHL: the Philippines.

j VNM: Vietnam.

k The number of cities enrolled in the Healthy Cities initiative in the respective country.

l GDP: gross domestic product.

m N/A: not available.

### Ethical Considerations

All components of this survey were approved by the IRB of Yonsei University in Korea (IRB 1041849-202401-SB-018-01). This study does not involve human subjects research; therefore, no compensation procedures were required, and there are no issues related to privacy or confidentiality. These aspects were thoroughly reviewed and confirmed by the Yonsei University IRB.

## Results

### AFHC Member Countries

Of the 9 AFHC member countries included in this study, the Republic of Korea had the largest number of healthy cities (109), followed by China (34) and Japan (31). China had the largest population (1410 million), followed by Japan (125.1 million) and the Philippines (115.5 million). Gross domestic product per capita was the highest in Australia (64,491), followed by Japan (33,815) and Korea (32,254). Life expectancy was the highest in the Republic of Korea and Japan (both 84 y). Access to electricity was the lowest in Cambodia (70%) with the remaining countries having 95% or higher access. The internet use rate was the highest in Malaysia (97.4%), followed by the Republic of Korea (97.2%), Australia (96.2%), and Japan (82.9%).

### SPIRIT Approach for the Digital Era

#### Setting and Sustainability (S)

Digital healthy cities were evaluated in terms of setting and sustainability. The findings indicated that Australia, Cambodia, and the Republic of Korea must ensure sustainability through telemedicine, while China should establish a digital healthy city database by integrating the public’s electronic medical records. Malaysia, Mongolia, and the Philippines need to sustain their digital healthy cities by establishing digital platform settings for communities (Table 2).

**Table 2.** Setting and sustainability (S) of the SPIRIT^a^ checklist by country.

Country	Content
Australia	Sustainability through telemedicine and digital health networking Sustainable digital healthy city through digital medical infrastructure
Cambodia	Provide high-quality medical service by addressing the digital medical disparity Expand sustainability by providing stable high-speed internet
China	Digital health database that integrates the public’s electronic medical records Health application, wearable, and digital health education
Japan	Sustainable urban planning through exemplary cases of digital healthy cities Policy decisions based on integration, education, and collaboration-focused data
The Republic of Korea	Establish a telemedicine system by digitalizing the existing health care facilities Establish systems to protect data and prevent leakage of personal information
Malaysia	Digital healthy city approach through digital technology and public health collaboration Secure data by strategically placing smart kiosks in the communities
Mongolia	Establish a digital healthy city hub through a digital platform Expand sustainability through digital health experts and digital coordinators
The Philippines	Digital healthy city with community participation Ensure sustainability by providing stable high-speed internet
Vietnam	Establish a sustainable digital healthy city by installing data protection measures Evidence-based digitally healthy city powered by research

a SPIRIT: setting approach and sustainability; political commitment, policy, and community participation; information and innovation; resources and research; infrastructure and intersectoral; and training.

#### Political Commitment, Policy, and Community Participation (P)

We examined political commitment, policy, and community participation in the context of digital healthy cities across 9 countries. The findings emphasized interdepartmental collaboration involving private organizations, schools, and civil institutions. Cambodia, China, and the Philippines referred to building digital healthy cities through community participation via digital platforms. Additionally, the Republic of Korea is implementing digital healthy cities by disclosing health-related public data (Table 3).

**Table 3.** Political commitment, policy, and participation (P) of the SPIRIT^a^ checklist by country.

Country	Content
Australia	Health in all policies (health care, environment, and education) Interdepartmental collaboration Competency skills training Data protection Monitoring
Cambodia	Digital leadership Interdepartmental collaboration Strengthen competency for digital health Community involvement (digital platform) Partnership with the technology industry
China	Community involvement (digital platform) Intensive training of promising talents in each department Centralized and comprehensive policy approach through digital healthy cities Comprehensive and encompassing digital healthy city framework
Japan	Health in All Policy approach Digital healthy city policies centered on transportation, education, housing, and digital infrastructure All departments demand to receive a health impact evaluation Health in All Policy approach by forming an interdepartmental committee
The Republic of Korea	Dedicated task force team for Health in All Policy approach Open data policy to allow access to health-related data Digital healthy city education for all personnel in charge of health-related matters in all departments Accelerate the transition to a healthy city by installing digital devices in communities
Malaysia	Establish digital health laboratories to make effective policies Share policies among various departments and set common health goals Devise digital healthy city policies to preserve the environment
Mongolia	Integration of various areas in digital healthy city policies Bolster responsibility of digitally healthy city policy makers Build public-private partnership
The Philippines	Propose digital healthy city policies through interspecialty collaboration, including health, transportation, education, environment, and information technology Digital healthy city through digital health platforms
Vietnam	Digital healthy city policy decisions based on data Feedback from digital healthy city experts every 2 years

a SPIRIT: setting approach and sustainability; political commitment, policy, and community participation; information and innovation; resources and research; infrastructure and intersectoral; and training.

#### Information and Innovation (I)

We analyzed digital healthy cities in terms of information and innovation. Australia has approached this from a data-centralization perspective. Cambodia has provided telemedicine services through digital health kiosks. China is using digital mental health platforms such as lifestyle fitness apps, green technology, and AI diagnostic tools. Japan is enhancing the digital-use competencies of older adults in their aged society and is planning innovative digital healthy cities based on their needs, such as promoting digital detox weekend campaigns. The Republic of Korea is realizing digital healthy cities with AI platforms for mental health counseling and integrated electronic medical record systems. Malaysia is installing digital kiosks where community residents can obtain instant health information, training community health professionals for this purpose. Mongolia has recognized the need to build digital healthy cities for nomadic communities by facilitating mobile health and telemedicine participation. The Philippines provides telemedicine consultations through the digital health access drive and aims to develop digital healthy cities based on health care big data. Vietnam is developing digital healthy cities based on data and the digital city profile (Table 4).

**Table 4.** Information and innovation (I) of the SPIRIT^a^ checklist by country.

Country	Content
Australia	Address urban noncommunicable diseases and mental health deterioration Develop public parks and bike lanes Require collaboration among various departments Digital healthy city through centralization (eg, data centralization)
Cambodia	Telemedicine service by implementing digital health kiosks Require increasing awareness for a healthy lifestyle Promote active participation of community members and analyze feedback Improve urban living environment through public-private collaboration
China	Digital mental health platform Lifestyle fitness app Green technology spaces Data-based AI^b^ diagnostic tool
Japan	Strengthen older adults’ digital competency Innovative digital healthy city planning using digital technology for older adults Digital detox weekends campaign
The Republic of Korea	Digital platform for AI mental health counseling Establish a real-time digital monitoring system for respiratory disorders Comprehensive electronic medical record system Digital program for walking through collaboration between Ministry of Health and Welfare and the Ministry of Land, Infrastructure, and Transport
Malaysia	Immediately acquire health information by installing digital kiosks in public areas Digital health education through community health care professionals Expand digital health by integrating nonhealth sectors, such as education and entertainment
Mongolia	Develop a digital healthy city system for nomadic communities Reduce health disparity by promoting mobile health and telemedicine for nomads Collaboration between the green healthy city and digital healthy city Develop mobile health care facilities
The Philippines	Provide telemedicine counseling and health education through the digital health access drive Develop and analyze the digital city profile Develop a digital healthy city based on health care big data
Vietnam	Inspect walking facilities through Geographic Information System Digital healthy city approach Digital healthy city based on the digital city profile

a SPIRIT: setting approach and sustainability; political commitment, policy, and community participation; information and innovation; resources and research; infrastructure and intersectoral; and training.

b AI: artificial intelligence.

#### Resources and Research (R)

We examined the funding and research for digitally healthy cities. Australia is analyzing the digital health gap and planning research on digital healthy cities in cooperation with the Australian Research Council. Cambodia evaluated its digital infrastructure and defined key performance indicators by mapping health priorities. China monitors health burden assessments and conducts research to enhance digital literacy while also studying the environment, policy, and regulations for digital healthy cities. Japan approaches digital healthy cities by analyzing digital health accessibility and identifying gaps. Additionally, they are conducting research on telemedicine platforms. The Republic of Korea aims to expand its digital healthy cities through the Korea Healthy City Partnership. The partnership seeks to develop key performance indicators and establish shared objectives for digital healthy cities. Mongolia is developing digital healthy cities with privacy-protected digital health regulations (Table 5).

**Table 5.** Resources and research (R) of the SPIRIT^a^ checklist by country.

Country	Content
Australia	Establish priority goals for a digital healthy city Analyze digital divide Health outcome metrics Joint resources investment by public and private sectors Research on digital healthy city through collaboration with the Australian Research Council
Cambodia	Networks of major stakeholders (eg, leaders, health care professionals, urban planning, and companies) Evaluate digital infrastructure (eg, internet, digital device use, and platform) Health priority mapping Define key performance indicators for a digitally healthy city
China	Health burden evaluation monitoring Research to improve digital literacy Foster a digital healthy city environment through sociocultural evaluation Research on policies and regulations for a digital healthy city
Japan	Develop a framework to evaluate the need for a digital healthy city Analyze digital health access among various demographic groups Analyze health disparities for a digital healthy city Research on artificial intelligence diagnosis and telemedicine platforms
The Republic of Korea	Expand digital healthy city through the Korea Healthy Cities Partnership Establish and share a digital healthy city database among cities Establish plans based on the latest literature and information on digital healthy city
Malaysia	Develop health initiatives to develop regions with poor digital infrastructure Analyze digital health disparities and implement a digital healthy city focused on vulnerable regions and groups Develop key performance indicators and establish common digital healthy city goals
Mongolia	Understand Mongolia’s legal digital health regulations and develop a digital healthy city that protects personal information
The Philippines	Foster experts through focus group interviews with various stakeholders Understand the digital health policy environment and innovate regulations Develop key performance indicators and establish common digital healthy city goals
Vietnam	Appropriate allocation of resources and funds through financial analysis, technical analysis, and risk assessment of digital healthy cities

a SPIRIT: setting approach and sustainability; political commitment, policy, and community participation; information and innovation; resources and research; infrastructure and intersectoral; and training.

#### Infrastructure and Intersectoral (I)

We examined the infrastructure and intersectoral measures for promoting digital healthy cities. Australia is strengthening the functions and capabilities of departments associated with digital healthy cities, requiring regular workshops for knowledge sharing. Cambodia and China are focusing on enhancing infrastructure through digital healthy city operation committees and collaboration with the Ministry of Health and Ministry of Urban Planning as well as other city administration departments. Japan is establishing a digital healthy city committee and uses local media and digital platforms for communication. The Republic of Korea is emphasizing building infrastructure for digital health companies and networking through digital healthy city conferences. The Philippines is developing infrastructure involving urban information and communication technology developers and coordinators to build networks of environmental experts (Table 6).

**Table 6.** Infrastructure and intersectoral (I) of the SPIRIT^a^ checklist by country.

Country	Content
Australia	Need to strengthen the functions and competencies of departments linked to digital healthy cities Sharing of knowledge and skills through periodic workshops Campaigning and promotions through online platforms and the internet
Cambodia	Bolster infrastructure through the activities of the digital healthy city operation committee Cambodia digital healthy city initiative operation committee needs to collaborate with the Ministry of Health, Ministry of Urban Planning, and city administrative bodies
China	Bolster infrastructure through the activities of the digital healthy city operation committee Collaboration among public agencies (Ministry of Health), private sector (information technology companies), hospital personnel (physicians and traditional Chinese medicine practitioners), academia (college faculty), and civil society (health nongovernmental organization)
Japan	Establish a digital healthy city committee with the Ministry of Health and local government leaders Communication through local media and digital platforms
The Republic of Korea	Establish various digital health company infrastructure around artificial intelligence conglomerates Expand the network around the Korea Healthy Cities Partnership
Malaysia	Establish a department dedicated to technological advancements for digital healthy cities Establish a digital healthy city network by hosting various digital healthy city conferences
Mongolia	Launch Mongolia digital healthy city partnerships Consider the expertise of the digital healthy city policy strategy team, operation, and research teams
The Philippines	Develop infrastructure involving information and communication technology developers and development coordinators Establish a network involving environmental experts to develop digital healthy cities
Vietnam	Establish and execute sustainable digital healthy city plans by developing professional project teams for a digital healthy city

a SPIRIT: setting approach and sustainability; political commitment, policy, and community participation; information and innovation; resources and research; infrastructure and intersectoral; and training.

#### Training (T)

We analyzed training initiatives for realizing digital healthy cities in each country. Australia regularly engages in education and research through exchanges with academic institutions. Cambodia requires settings, such as a digital healthy city initiative conference and the Global Health Summit (Table 7).

**Table 7.** Training (T) of the SPIRIT^a^ checklist by country.

Country	Content
Australia	Educate digital healthy city-related personnel through periodic workshops Education and research through exchanges with academic institutions
Cambodia	Cambodia digital healthy city initiative conference Need digital healthy city Asia conference Global health summit setting
China	Healthy China 2030 conference Host a digital healthy city forum Smart and healthy city exhibition
Japan	Host conference with the involvement of Japan Smart Community Alliance Education using digital kiosks Education using community fitness apps
The Republic of Korea	Provide public health promotion workshops focused on digital health Expand digital healthy city through the Korea digital health association Organize digital health master class
Malaysia	Host Digital Health Malaysia to invite healthy cities in nearby countries for a joint education on digital healthy city planning and directions Host smart city conference
Mongolia	Education and training on digital innovation in health care Organize Mongolian Smart City Association
The Philippines	Host an international digital healthy city forum through the Digital Health Philippines Summit Need collaboration through digital healthy city sister city agreements
Vietnam	Actively promote digital healthy cities through social media Research on health technologies and collaboration

a SPIRIT: setting approach and sustainability, political commitment, policy, and community participation, information and innovation, resources and research, infrastructure and intersectoral, and training.

## Discussion

### Principal Results

This study analyzed the digital capabilities of AFHC member countries amid the ongoing transition toward a digital era using the AFHC SPIRIT checklist with the *openai* library in Python and the ChatGPT (version 4.0) large language model. The following implications were obtained.

First, it is necessary to prepare for digital healthy cities by forming digital healthy city networks and committees to promote sustainability and infrastructure. Specifically, an interdepartmental committee should be established to facilitate collaboration among health city-related departments. Further, a holistic “Healthy in All Policy during digital era” approach should be adopted. The AFHC should enhance intercity networking in the digital era during its biennial general meetings. Additionally, it is important to create a framework based on an in-depth understanding of social dynamics, policy structures, governance networks, and urban infrastructure [20].

Second, as digital healthy cities increasingly rely on data-driven policies, the importance of data protection and personal information security has been highlighted. With the volume of daily generated data expanding worldwide, the era of big data is imminent. In such an era, personal information protection is a significant social issue [21]. This study found that data-based policy decisions are crucial for digital healthy cities and requires reviewing and analyzing various data types. The increasing use of community residents’ data necessitates diverse protective measures, such as legal regulations and training for responsible personnel. For instance, some healthy cities have adopted AI care robots to provide home-based care services for older adults. As all information about the client is collected through these care robots, heightened precautions must be taken to protect personal information [22].

Third, there is a need for cooperation between various organizations and sectors, such as public agencies, industries, schools, hospitals, and private organizations, to build a digital healthy city network and implement intersectoral healthy city policies. Such cooperation is crucial to promote digital healthy cities. It is particularly important to establish an infrastructure for community health management by linking digital information technology and AI-related industries with school research institutions and including public institutions, hospitals, and private organizations. Developing a shared platform across these sectors is essential to systematically implement digital healthy cities initiatives [7].

Fourth, there is a need for digital healthy city policies tailored to each country’s unique digital capabilities, characteristics, policies, and cultures. The WHO reviewed the focus group analyses of healthy cities worldwide and emphasized the importance of developing appropriate policies for each country and city, defining this as the “glocal approach” [23]. With advancements in digital technology, there is a growing focus on personalized medicine and public health. Accordingly, digital healthy city policies must be customized, considering the diverse characteristics of each country.

Finally, this study highlights the importance of education for key groups in digital healthy cities—policy makers, civil servants, health care providers, and residents. As digital transformation gains traction worldwide, AFHC member countries are also witnessing accelerated digitalization. However, the digital divide between cities, genders, and socioeconomic classes in these countries is widening [24]. The WHO cautions that, without adequate digital literacy among managers and users, the efficacy of digital technologies remains limited even after commercialization [10]. Telemedicine, digital health kiosks, and data management systems are key components of digital healthy cities. To use these services as intended, competency education is essential. Most importantly, digital literacy education for older adults is crucial, given the rapid pace of population aging [25,26].

### Limitations

This study has a few limitations. First, as ChatGPT (version 4.0) is trained only until April 2023, the latest developments were not reflected in our survey. Second, the number of healthy cities varied across countries. Third, the SPIRIT checklist was interpreted by only 3 experts. While objective assessments were performed based on their expertise, the number of experts may not have been sufficient for subjective results. Thus, future studies should involve at least 5 experts. Fourth, owing to the proprietary nature of OpenAI’s ChatGPT large language model, its full workings and application in this study could not be completely explained. Additionally, the differing levels of political commitment and resources among countries complicate comparisons. Wealthier nations such as South Korea and Japan invest heavily in digital health, while countries such as Cambodia and Mongolia focus on basic health care needs. Tailored strategies are needed to account for these economic and health care disparities, and future studies should consider long-term progress in countries with less developed infrastructures.

### Conclusions

This study analyzed the digital health city capabilities of 9 AFHC member countries in the digital era. The findings suggested that these countries must prepare to attain sustainable digital healthy cities by establishing networks and committees. Additionally, digital healthy cities develop big data-driven policies, and this requires systems to ensure personal information protection. This study also emphasized the need for collaborative initiatives across various sectors to systematically implement digital healthy cities. Finally, digital healthy cities are expected to be less effective even with good policies and environments if users have poor digital literacy; thus, policies for improving digital literacy among both managers and users will be required.

## Abbreviations

AFHC: Alliance for Healthy Cities
AI: artificial intelligence
IRB: institutional review board
SPIRIT: setting approach and sustainability; political commitment, policy, and community participation; information and innovation; resources and research; infrastructure and intersectoral; and training
WHO: World Health Organization
